# Assessing Self-Concept in Children (Aged 5–7) with Functional Dyslalia

**DOI:** 10.3390/children10071238

**Published:** 2023-07-18

**Authors:** Isabel Angustias Gómez Pérez, Carmen del Pilar Gallardo-Montes, Julio Ballesta-Claver, Mᵃ Fernanda Ayllón Blanco

**Affiliations:** 1 La Inmaculada Teaching Center (LITC), Evolutionary Psychology and Education Department, University of Granada, 18013 Granada, Spain; isabelgomez@cmli.es; 2 Department of Didactics and School Organization, University of Granada, 18071 Granada, Spain; 3 La Inmaculada Teaching Center (LITC), Didactics of Experimental Sciences Department, University of Granada, 18013 Granada, Spain; juliosci@cmli.es (J.B.-C.); mayllonblanco@cmli.es (M.F.A.B.)

**Keywords:** self-concept, dyslalia, children, specific learning disabilities

## Abstract

Language not only plays a powerful role in human life, as it is also a crucial factor in our minds. It shapes our personality, memory and even the way in which we see the world, as well as playing a fundamental role in the building of self-concept and self-esteem. Having a good self-concept, that is, knowing one’s own qualities and strengths, will, in turn, promote good self-esteem. The aim of this research was to analyze self-concept in 50 children (aged 5–7) with functional dyslalia in the city of Granada (Spain). A quantitative approach was taken, with a non-experimental design; it was descriptive, cross-sectional and correlational. The Perception of Child Self-concept Scale (PCS), a Spanish scale, was used. In general, the children who were interviewed showed a medium level of self-concept. It is noteworthy that differences were found in the average scores on the scale according to the sex of the children, with girls showing a higher level of self-concept than boys. Participants scored higher on Factor 1, *family attachment*, followed by Factor 3, *feelings*, with the values of both these factors decreasing with age. On the other hand, lower average scores were found for Factor 2, *environment*, and Factor 4, *autonomy*. It was found that self-concept was higher in young children (five-year-old children) as well as in those who studied in rural areas. Finally, guidelines for improvement were provided. Self-concept is a fundamental aspect of personality, but it is not innate; it develops and evolves.

## 1. Introduction

Communication plays a vital role in life. It is an essential aspect of relationships, personal development, identity and social interaction [1]. Therefore, language is quintessentially human behavior. It is the main tool for communication [2], and it is the way to develop our thinking, to grasp and understand reality, to establish social relationships with others and to control our own behavior and that of other people [3]. For these reasons, schools, especially in Infant and Primary Education, must pay special attention to children’s language and carefully follow its developmental process. However, in order to be able to give this special attention, a sound knowledge of how children acquire language and how they develop their linguistic abilities is needed.

It could be said that speech, a valuable vehicle in human development, has a direct impact on language development. Therefore, maximizing language capacity during Infant Education, before the child enters the Primary Education stage, should be considered as a primary goal [4]. In this way, the possible negative effects that articulation difficulties may have on a child’s performance at school and on their self-concept would be avoided. Functional dyslalia [5] is one of the most common speech disorders in children. It is a “change in the pronunciation of one or more phonemes by alteration, substitution or omission in people who do not have central neurological lesions or malformations of the phonoarticulatory organs” [6]. This disorder often stems from poor oral habits, such as the incorrect positioning of the tongue against or between the front teeth [7]. However, several authors [8,9,10] have reported that other habits, such as prolonged bottle and dummy use, thumb or lip sucking, nail biting, bruxism or type of feeding, can also cause dyslalia.

This type of speech disorder can have a negative effect on children who suffer from it; it has a negative impact on their social, emotional and functional well-being [11,12]. Previous studies such as Hassan’s [13] showed that a high percentage of children with dyslalia had behavioral problems. Even in Cantwell and Baker’s research [14], the authors noted emotional and behavioral instability in children with this disorder. Therefore, early intervention is essential [15], given that the prevalence of this speech disorder in Spanish children aged 4 to 7 is high, standing at around 50% [16].

A child who feels different from others in their speech will develop a growing sense of insecurity. This will make them withdraw into themself for fear of speaking and being treated by others in a way that makes them feel inferior. As a result, their self-confidence and possibilities for improvement could be diminished.

Who am I? This is one of the most important questions a human being asks. “Self-concept” comes into play when this question is answered. It refers to how we feel about ourselves, and it is based on our knowledge of what we have been and what we have done. Its function is to guide and determine what we will be and what we will do in the future. Therefore, self-concept helps us both to understand ourselves and to control or regulate how we behave [17]. A high level of self-concept allows children to adapt socially to different situations, it provides mutual respect and social support and reduces attitudes of shyness and loneliness [18]. Previous studies, such as Makri and Giannouli [19], investigated precisely how cultural experiences, such as socialization or language acquisition, influence the way we process information, determining the way we interact with our environment.

Self-concept emerges from the child’s environmental experiences, both at home and in interactions with others (parents or friends). In view of its developmental nature, the assessment and measurement of self-concept can be somewhat problematic [20]. The importance of self-concept during the period of Infant Education and the identified positive relationships that prove the need to promote its development at this age are emphasized by authors, such as Garaigordobil and Berrueco [21]. According to these authors, it is important to implement psycho-pedagogical intervention programs aimed at enhancing self-concept, as this acts as a preventive measure by boosting children’s self-confidence and fostering their ability to regulate their own behavior.

In relation to the above, different studies have investigated the levels of self-concept in pupils in Infant and Primary Education [21,22,23,24,25,26]. Cámara-Martínez et al. [27] discovered that the implementation of active teaching programs using playful mathematical games significantly increases both self-concept and personal and academic self-esteem in pre-school children. They used the Perception of Child Self-concept Scale (PCS) by Villa and Azumendi [28], a scale called the “Percepción del Autoconcepto Infantil” (PAI) in Spanish. First, these authors reported that girls are more likely than boys to improve their personal and academic self-esteem. Subsequently, Aparicio and Alcaide [29] used the same instrument [28] to study self-concept in young children between the ages of 3 and 6. They concluded that boys had a better self-concept than girls. Similarly, they found statistically significant differences in the *feelings* dimension, where boys also stood out. This suggests the need to study the dimensions of the test separately to see if there are significant differences that might affect the final result. On the other hand, it is also useful to examine other variables that may influence self-concept, similar to Zavala and Magallanes [30]. They analyzed the relationship between self-concept and academic achievement in the school subject of Language in 5- and 6-year-old pupils, using the Pictorial Scale of Perceived Competence and Social Acceptance for Young Children (PSCA) [31]. It was concluded that the level of self-concept has no impact on academic performance.

Several studies have also investigated the relationship between Specific Learning Disabilities (SLDs) [32] and self-concept. Such studies have considered dyslexia and its relationship with self-esteem and mental health [33]; attention deficit hyperactivity disorder (ADHD) and its impact on positive illusory bias [34]; or self-efficacy in children with dysphemia [35].

However, there are no studies focusing on how functional dyslalia affects self-concept during Infant and Primary Education. Therefore, the objectives of this research are:

1. To assess self-concept in children (aged 5 to 7) with functional dyslalia.
2. To analyze whether there are any correlations between the dimensions of the PCS (factors) and the different variables, such as sex, level of education, age and type of center.
3. To determine whether there are statistically significant differences according to sex, level of education, age and type of center.

## 2. Materials and Methods

The study was carried out using a quantitative approach. The design was non-experimental; it was descriptive, cross-sectional and correlational.

### 2.1. Participants and Procedure

The sample was obtained through an initial screening of 650 pupils in the last year of Infant Education (5 year olds) and the first year of Primary Education (6–7 year olds). This choice of ages is justified by the fact that the process of acquiring the phonological inventory culminates at this age [36]. The pupils were enrolled in six different schools, randomly selected, in the city of Granada; three were urban schools and the other three were in rural areas. Each school had 2 or 3 parallel classes for each age group/level.

Next, in order to identify which children were “making mistakes when speaking”, the teachers carried out an observational protocol referred to in Gallego Ortega [37] to detect language problems in the classroom. As a result, 72 children were selected. These pupils were evaluated individually by professionals using standardized and non-standardized tests, always in appropriate places and outside the classroom, both in the initial and final evaluation phases. The non-standardized tests used for the initial assessment of each child’s articulatory ability were [38]: spontaneous verbal production, elicited verbal production and imitation. These tests were not designed to obtain new information but were carried out as a second screening to complete the initial one conducted by the teachers of the Infant (5 year olds) and Primary School children (6–7 year olds). Similarly, because of the obvious relationship between verbal expression, breathing and hearing, each child’s respiratory system was assessed using the Glatzel and Rosenthal tests, which are well known among professionals. The pupils’ hearing was assessed using the MAICO Pilot Hearing Test.

The standardized test used to evaluate the pupils’ phonological performance was the Induced Phonological Recording [36]. This test material comprises 57 cards designed for phonological assessment in induced expression and repetition, with a scale for children between 3 and 7 years old. This test definitively showed which phonemes of the Spanish language (/l/, /θ/, /s/, /r/, /$\bar{r}$/), diphthongs (io, ie, oa, ei, au, ue, ia) and syphons (CLV, CRV) the children did not articulate correctly, as well as the type of error they made (substitution, omission, distortion and insertion).

Finally, pupils with intellectual or sensory disabilities or with organic problems were excluded from the sample [39]. This excluded 22 pupils, leaving those who only had difficulty articulating speech, which was a total of 50 pupils, which represents 7.7% of the total sample. All this was carried out with the written consent of the parents, by sending the families a document to sign, in accordance with the data protection law. In this sample, 32 pupils were boys (64%) and 18 were girls (36%), all aged between 5 and 7 years old (*M* = 5.88; *SD* = 0.895). They were enrolled in Infant Education (40%) and Primary Education (60%) and came from urban (56%) and rural (44%) schools.

After interviewing the pupils in order to confirm the existence of functional dyslalia, an investigation was carried out to find out about the articulatory language development and self-concept of pupils in the second stage of Infant Education and the first stage of Primary Education, which is the main goal of this paper. These pupils were assessed for self-concept using the PCS (Perception of Child Self-concept Scale).

### 2.2. Instrument

In this work, the Perception of Child Self-concept Scale (PCS) [28], which is based on a Likert scale, was used to assess self-concept in 50 Infant and Primary School pupils with functional dyslalia. It used drawings of situations in which several children are engaged in some activity, whether at school, at a birthday party, etc.

This scale contains 34 items, grouped into 10 dimensions [40]. These are: *Autonomy*: feeling of independence; *Self-worth*: feeling of one’s own competence; *Awareness of possession*: of friends, of objects, etc.; *Security*: confidence in accomplishing tasks; *Family*: how one feels about family relationships; *Feelings*: general mood (sad, happy, etc.); *Physical aspect*: physical appearance; *Sport*: worthiness in competition; *Classroom:* the child in the school world; *Social:* the child’s social relationships. The scores obtained in each dimension provide a total score that indicates the child’s global level of self-concept.

In addition, an exploratory factor analysis of the questionnaire was achieved, using the selected sample of pupils, by means of a Likert-type scale (ordinal scale from 1 to 5). The open-source statistical program R (version 4.2.2) from Rstudio (version 2023.03.0) was used. Kaiser–Meyer–Olkin factorial sampling adequacy (KMO) was utilized, which correlates pairs of variables [41] and generates a moderate value of 0.61, obtaining a value of 113.14 (*p* < 0.001) for Barlett’s test of sphericity. These data assume that responses are substantially related to one another, resulting in clusters of items with the possibility of the generation of different factors. When the exploratory factor analysis was performed, a total of four factors were obtained using the Kaiser–Guttman criteria (eigenvalues greater than 1) [42]. Varimax rotation with Kaiser normalization [43,44] was used to obtain conceptually similar and significant clusters of issues of the variables [40], explaining 67.4% of variance (Table 1). The four factors that were obtained from among the 10 dimensions were categorized as follows: (1) Factor 1: *family attachment*; (2) Factor 2: *environment*; (3) Factor 3: *feelings*; (4) Factor 4: *autonomy*.

The reliability of the results was guaranteed by internal consistency using Cronbach’s alpha criterion [45,46,47]. The alpha value gave an acceptable result of α = 0.63. On the other hand, since a moderate scale of response options was used, as well as a small sample size, it is possible to use the McDonald omega factor, which is based on communalities and on the estimation of factor loadings [45,46,47]. Thus, a moderate reliability value of ω = 0.73 was obtained.

### 2.3. Data Analysis

After recording the obtained results, appropriate statistical analyses were carried out in order to determine whether children with functional dyslalia have a level of self-concept that could be detrimental to their development. The following data were analyzed with the statistical package IBM SPSS software v.26.0 for Windows, provided by the University of Granada, Spain. Descriptive statistics (mean, standard deviation, minimum and maximum) were calculated. Non-parametric inferential analysis and intrafactorial correlation analysis were carried out. For the analysis of correlations, Spearman’s correlation coefficient was calculated [48,49], and to examine the comparisons between “sex”, “educational level” and “type of school”, the *U*-Mann–Whitney non-parametric test was implemented, since the data did not show a normal distribution (Shapiro–Wilk < 0.05). The effect size was also estimated using Cohen’s *d* calculation [50] or r factor [51]. For the variable “age”, the Kruskal–Wallis test and the consequent Games–Howell post hoc test were applied, and the effect size was also obtained using Hedges’ *g* [52].

## 3. Results

PCS scores, based on the scores obtained on the Likert scale used (from 1 to 5), indicated that the children had a medium level of self-concept (*M* = 3.27; *SD* = 0.54) (see Table 2). Here, it can be observed that the girls’ self-concept was slightly higher than that of the boys, and when the Mann–Whitney U test for independent samples was used, a medium effect size was obtained (*U* = 239.5, *p* = 0.03, *r* = 0.30), with the girls showing a medium–high self-concept compared to boys’ medium self-concept [*M*_girls_ = 3.42 (medium-high); *M*_boys_ = 3.18 (medium)]. There is a tendency for girls to improve their self-concept with age: 5 years (*N* = 9), 3.43 ± 0.55; 6 years (*N* = 3), 2.93 ± 0.91; 7 years (*N* = 6), 3.65 ± 0.42; on the contrary, boys self-concept decreases: 5 years (*N* = 14), 3.47 ± 0.27; 6 years (*N* = 7), 3.31 ± 0.50; 7 years (*N* = 11), 2.86 ± 0.56. Although the other results were not significant in comparison, it is noteworthy that the 5-year-old children from Infant Education also had a higher self-concept than the other participants.

In addition, the children from urban centers showed a slightly higher level of self-concept than the children from rural centers (Table 2).

The descriptive results for the four factors resulting from the PCS scale are presented in Table 3. In general, the responses of the participants reached higher values in Factor 1 (*family attachment*) followed by Factor 3 (*feelings*), which shows that the children achieved better results in aspects related to their family environment, physical aspect, feelings and emotional state. However, lower mean scores were found for Factor 2 (*environment*) and Factor 4 (*autonomy*). This means that they had lower perceptions regarding their feelings of security and self-confidence, their relationships in the classroom and with peers, their own autonomy, their personal self-worth and their physical competence.

The correlation analysis (Table 4) showed that there were positive and significant correlations. As expected, the correlation between *age* and *educational level* was high (*p* < 0.01), whereas the correlations related to the *type of center* and *age* and between *type of center* and *autonomy* (Factor 4) were weak (*p* < 0.05), as was the correlation between *family attachment* (Factor 1) and *feelings* (Factor 3).

Negative and weak, but nonetheless significant, correlations were also observed between *family attachment* (Factor 1) and *age* (*p* < 0.01), and between *family attachment* and *educational level* (*p* < 0.05). Similarly, there were correlations between Factor 3 (*feelings*), *age*, and *educational level*.

The inferential analyses regarding the different variables and resulting factors revealed differences in the variables, with the exception of *sex*, where no differences were found, as the correlation study also shows.

The *age* variable showed statistically significant differences in only two of the four factors (see Table 5). The majority of the group of 5-year-old pupils showed higher scores for Factor 1 (*family attachment*), in comparison to the group of 7-year-old pupils. The Games–Howell post hoc test was used and it showed a small effect size, obtaining a Hedges’ g-value of *ε*^2^ = 0.017.

The same group (5 year olds) also scored higher on Factor 3 (*feelings*) compared to the 6-year-old pupils, showing a small effect size (*ε*^2^ = 0.026).

According to the *type of center* (*U* = 189.50; *p* = 0.019; *d* = 0.67), pupils from rural areas scored higher on Factor 4 (*autonomy*) (*M*_rural_ = 3.04; *SD*_rural_ = 0.79) than those from urban centers (*M*_urban_ = 2.54; *SD*_urban_ = 0.69), resulting in a medium effect size (*d* > 0.50).

The *level of education* also gave significant results (*U* = 185.00; *p* = 0.018; *d* = 0.77). Pupils in Primary Education obtained lower scores for Factor 3, *feelings* (*M*_primary_ = 3.20; *SD*_primary_ = 1.16), compared to pupils in Infant Education (*M*_infant_ = 3.95; *SD*_infant_ = 0.76), data that were consistent with the results obtained with the *age* variable, obtaining a medium effect size (*d* > 0.50).

## 4. Discussion

A child’s self-esteem will consequently have an impact on their social, emotional and intellectual behavior. A lack of self-esteem significantly influences mental well-being, satisfaction, health and also psychological functioning. Therefore, according to Villa and Auzmendi [28], having an adequate self-concept influences both oneself and others.

Self-concept not only affects behavior but also the way certain elements of the world are perceived. Our level of self-concept influences how we perceive and experiment with our environment, that is, how much importance we give to different experiences.

Self-concept also has an impact on others. If a person has a good self-concept, they are able to accept the experiences of their life, their perceptions are not distorted, there is no discrepancy between their real self and their ideal self, they have less defensive attitudes, they perceive reality more authentically and they accept the reality of others. This is why self-concept is a fundamental factor of personality. As we have seen, self-concept is not innate; it develops and evolves, which explains the importance of measuring and assessing it from early childhood [28].

Language is not only a great force in a child’s life but also a determining factor in a child’s mind, as explained in detail throughout this work. It has an impact on a child’s personality, memory and even on their perception of the world. It is in harmony with a multitude of variables, but, when focusing on dyslalia, it can be seen that children with this speech disorder form their self-concept around it and, as a consequence, their self-esteem is also gradually affected.

With regard to the first objective of this study, assessing self-concept in children with functional dyslalia, it was observed that pupils with this speech disorder showed a medium level of self-concept (*M* = 3.27), according to the data provided by the PCS scale. It is difficult to compare this information with previous research since the existing literature has not yet studied the level of self-concept in pupils with functional dyslalia and its effects. However, when these data are compared with the results obtained for the pupils without this speech disorder [24,27,40], it can be observed that, in all cases, the usual level of self-concept for this age group is medium–high, which means that the effect of functional dyslalia is to lower self-concept by half a degree.

With regard to the second objective, which is the analysis of the existence or not of correlations between the different variables and factors, it was established that there is a significant relationship between *type of center* and *autonomy*. Pupils from rural centers showed higher levels of self-concept for Factor 4 (*autonomy*) than those from urban areas. This suggests that these pupils may perceive themselves better in terms of their sense of independence and autonomy, their sense of security and self-confidence and their sense of self-worth, as well as their feeling of self-competence. A study carried out by Jiménez Boraita et al. [53] between rural and urban areas of a region in the north of Spain can explain these attitudes. They found that the sense of belonging to the school is greater in rural areas, leading to greater social acceptance, which reduces both problems among classmates and the frequency of intra-family conflicts. Relationships were also found between the factor of *feelings* and *family attachment*. It can be concluded that there is a link between social interaction and the child’s relationship with their feelings, moods and schooling.

Regarding the third objective, which is to determine whether there are statistically significant differences between the variables that were analyzed globally, it is noteworthy that *sex* showed significant differences. In a study carried out by Aparicio and Alcaide [29], self-concept was assessed in children aged 3 to 6 without Specific Learning Disabilities. They found that boys had a better self-concept than girls. In our study of children with functional dyslalia, we found that girls scored slightly higher than boys. In this regard, it is worth mentioning the findings of Cámara-Martínez et al. [27], which are in line with our results. They indicate that girls were more likely to improve their personal and academic self-esteem by performing different activities over time than boys were. In our study, there was a tendency for girls to improve their self-concept with age.

The 5-year-old pupils showed higher scores for Factor 1 (*family attachment*) and Factor 3 (*feelings*), compared to 7-year-old pupils. Self-concept is a reflection of oneself that develops by interacting and comparing oneself with one’s peers [54]. This means that younger children have better self-perceptions in aspects related to themselves and their relationship with the school environment, in the affective and emotional environment, in terms of their relationships and their social context and in terms of their personal worth in sporting and competitive environments, as Jiménez Boraita et al. [53] also affirmed.

With regard to the level of education, pupils with dyslalia in Primary Education showed lower scores for Factor 3 (*feelings*) than pupils in Infant Education. This could suggest that pupils with dyslalia from higher educational levels, due to their higher evolutionary level and greater level of maturity, perceived themselves as more insecure in social relationships due to poor adjustment to the environment and higher levels of emotional and psycho-social problems, as Boerrigter et al. indicated [54].

The limitations of this research are mainly related to the sample and the context, given that only 50 children from Granada were included. As a prospective research project, and as a possible solution to the sample limitation described above, it would be interesting to extend the research to other cities in the autonomous community of Andalusia or even to other Spanish provinces. Similarly, it would be enriching to implement an intervention program based not only on the treatment of dyslalia but also on improvements in self-concept throughout the Primary Education stage. To this end, given the benefits of ICTs, such a program could include digital resources, like those used in Bernal et al. [55], Nevárez et al. [56], Tenesaca et al. [57] or Velázquez et al. [58].

## 5. Conclusions

Speech and language disorders often constitute a hidden disability that affects children’s behavior, social interactions and academic performance in school [12], which, in turn, affects their self-concept. This work attempts to contribute to this field, a field which has not yet been studied in depth for this age group. Using a quantitative study with a non-experimental design, we found out which variables and dimensions included in the Perception of Child Self-concept Scale (PCS) [19], grouped in factors, affect self-concept in pupils aged between 5 and 7 who have been diagnosed with functional dyslalia. The results prove that children with functional dyslalia have a reduced level of self-concept, at least at a medium level according to this scale, in comparison with children who do not have it (medium–high level), with the difference that girls improve their self-concept more quickly with age or by carrying out activities for its improvement than boys [18].

Regarding the first objective of this research, it can be seen that the self-concept of children with functional dyslalia decreases with age. As the child grows, their family attachment decreases. Therefore, it is necessary to work on this interaction in Primary Education, strengthening the socio-affective relationships between pupils and their family [9].

With respect to the second and third objectives, no differences were found between the variables concerning the sex of the pupils. The autonomy of the pupil depends on the type of center in which they are enrolled. A rural center has the advantage of being close to home, as well as providing a familiar and pleasant personal environment [53]. All this will improve pupils’ self-concept and, therefore, urban centers should also strive to obtain these qualities. As far as family attachment is concerned, it creates good feelings in the pupils. These feelings decrease with age and affect self-concept. Therefore, more activities with parents are needed, and parents should be encouraged to reward their children for making an effort to improve their speech.

In conclusion, there is a need for more studies on functional dyslalia and self-concept in schools. This work aims to promote further research that will lead to more specific solutions that will not only help to improve self-concept but will also have positive repercussions on the pupils’ self-esteem and academic performance. A greater knowledge of pupils’ strengths and weaknesses will better equip us to create suitable improvement programs for the future.

## Figures and Tables

**Table 1:** Characteristic values obtained via exploratory factor analysis of the instrument.

	Factor 1 (*Family Attachment*)	Factor 2 (*Environment*)	Factor 3 (*Feelings*)	Factor 4 (*Autonomy*)
Dimensions	3, 5, 7	4, 9, 10	6	1, 2, 8
% Variance	26.9	17.8	12.1	11.0
% Accumulated	26.9	44.7	56.7	67.4
Kaiser-Guttman criteria (Characteristic values > 1)	1.64	1.33	1.09	1.03

**Table 2:** Descriptive statistics for the self-concept scores.

Variables		*M*	*SD*	*SCL*
Global self-concept (*N* = 50)		3.27	0.54	M
Sex *	Boys (*n* = 32)	3.18	0.49	M
	Girls (*n* = 18)	3.42	0.59	M-H
Educational level	Infant Education (*n* = 20)	3.38	0.42	M-H
	Primary Education (*n* = 30)	3.20	0.60	M
Age	5 years (*n* = 23)	3.40	0.39	M-H
	6 years (*n* = 10)	3.20	0.62	M
	7 years (*n* = 17)	3.14	0.64	M
Type of center	Urban (*n* = 28)	3.31	0.45	M-H
	Rural (*n* = 22)	3.22	0.64	M

Note. *M* = Mean; *SD* = Standard Deviation; *SCL* = Self-Concept Level obtained on the PCS, scaled according to the following criteria: 0–1.3 = Low; 1.3–2.3 = Medium-low; 2.3–3.3 = Medium; 3.3–4.3 = Medium-high; 4.3–5 = High. In this case, the predominant level of general self-concept is medium (M). However, there are four variables in which the level of general self-concept is medium-high (M-H). These are: sex (girls); educational level (Infant Education); age (5 year olds) and type of center (urban). Comparison tests: U-Mann–Whitney and Kruskal–Wallis tests. * The correlation is significant at level 0.05 (two-tailed).

**Table 3:** Minimum, maximum, mean and standard deviation of the scores obtained for each factor on self-concept (*N* = 50).

Factor	Minimum	Maximum	*M*	*SD*
Factor 1. Family attachment	2.00	5.00	3.85	1.02
Factor 2. Environment	1.33	4.33	3.12	0.76
Factor 3. Feelings	1.00	5.00	3.50	1.07
Factor 4. Autonomy	1.00	4.33	2.76	0.77

Note. *M* = Mean; *SD* = Standard deviation.

**Table 4:** Pearson correlation analysis regarding *sex*, *educational level*, *age* and *type of center* together with the four factors.

Variables	Sex	Educational Level	Age	Type of Center	F1	F2	F3	F4
1. Sex	1							
2. Educational level	−0.068	1						
3. Age	−0.039	0.811 **	1					
4. Type of center	0.259	0.066	0.347 *	1				
5. Factor 1. Family attachment	0.081	−0.307 *	−0.369 **	−0.203	1			
6. Factor 2. Environment	0.140	0.095	−0.039	−0.196	0.188	1		
7. Factor 3. Feelings	0.274	−0.346 *	−0.361 *	−0.114	0.329 *	0.143	1	
8. Factor 4. Autonomy	0.127	0.082	0.194	0.332 *	0.015	0.210	−0.049	1

Note. * The correlation is significant at level 0.05 (two-tailed); ** The correlation is significant at level 0.01 (two-tailed). F1 (factor 1); F2 (factor 2); F3 (factor 3); F4 (factor 4).

**Table 5:** Significant differences according to the participants’ age.

Factor	5 Year Olds (*n* = 23)		6 Year Olds (*n* = 10)		7 Year Olds (*n* = 14)		Kruskal-Wallis Test	
	*M*	*SD*	*M*	*SD*	*M*	*SD*	*K*	*p*
1. Family attachment	4.32	0.788	3.37	1.05	3.51	1.07	8.44	0.015 *
3. Feelings	4.04	0.77	2.70	0.67	3.23	1.25	12.76	0.002 **

Note. *n* = number of elements that make up the sample; *M* = Mean; *SD* = Standard Deviation; *K =* Statistic associated with the Kruskal Wallis test; *p* = Probability associated with *K*; Statistically significant: * *p* < 0.05; ** *p* < 0.01.

## Data Availability

Not applicable.

## References

[1] Srinivasaraghavan K., Pranav V., Radhakrishnan P. (2022). Impact of communication difficulty on the quality of life in individuals with Parkinson’s disease. Ann. Mov. Disord..

[2] Granada-Azcárraga M., Cáceres F., Pomés M., Ibáñez A. (2023). Trastorno específico del lenguaje y retraso de lenguaje: Un estudio sobre matrículas en escuelas de lenguaje en Chile. Rev. Electrónica Educ..

[3] Larroca H.D. (2023). Componente semántico en niños de edad preescolar con dificultad de lenguaje en las escuelas del Perú. Telos Rev. Estud. Interdiscip. Cienc. Soc..

[4] Pérez M.B., Tramadillo C.P., Peñafiel V. (2020). La estimulación temprana en el desarrollo de habilidades y destrezas del lenguaje en niños de educación inicial. Rev. Didasc@lia.

[5] Conde P., Quirós P., Conde M.J., Bartolomé M.T. (2014). Perfil neuropsicológico de niños con dislalias: Alteraciones mnésicas y atencionales. An. Psicol..

[6] Campos A.D., Campos L.D. (2014). Patologías de la comunicación. Proyecto docente para enfermería infantil. Dislalias. Enfermería Glob..

[7] Proffit W.R., Mason R.M. (1975). Myofunctional therapy for tongue-thrusting: Background and recommendations. J. Am. Dent. Assoc..

[8] Barbosa C., Vasquez S., Parada M.A., Gonzalez J.C.V., Jackson C., Yanez N.D., Gelaye B., Fitzpatrick A.L. (2009). The relationship of bottle feeding and other sucking behaviors with speech disorder in Patagonian preschoolers. BMC Pediatr..

[9] Farronato G., Giannini L., Riva R., Galbiati G., Maspero C. (2012). Correlations between malocclusions and dyslalias. Eur. J. Paediatr. Dent..

[10] Ferriolli B.H.V.M. (2010). Associação entre as alterações de alimentação infantil e distúrbios de fala e linguagem. Rev. CEFAC.

[11] Nagaram S.K., Maloji S., Kasiprasad M. (2019). Misarticulated /r/-Speech Corpus and Automatic Recognition Technique. Int. J. Recent Technol. Eng..

[12] Swain S.K., Sahu M.C., Choudhury J. (2018). Speech disorders in children: Our experience in a tertiary care teaching hospital in eastern India. Pediatr. Pol..

[13] Hassan E., Darweesh A., Ibrahim R., Zareh W. (2020). Psychological status of school-aged children and adolescents with dyslalia. J. Curr. Med. Res. Pract..

[14] Cantwell D.P., Baker L. (1991). Psychiatric and Developmental Disorders in Children with Communication Disorder.

[15] Moreira C., García Y., Amaro N.L. (2023). La gestión orientadora hacia la correcta pronunciación de los sonidos [-s] y [-r] en posición distensiva. Sinerg. Académica.

[16] Rey O.A., Sánchez-Delgado P., Palmer M.R.S., De Anda M.C.O., Gallardo V.P. (2022). Exploratory Study on the Prevalence of Speech Sound Disorders in a Group of Valencian School Students Belonging to 3rd Grade of Infant School and 1st Grade of Primary School. Psicol. Educ..

[17] González J.A., Núñez J.C., González S., García M. (1997). Autoconcepto, autoestima y aprendizaje escolar. Psicothema.

[18] Pérez J.I., Garaigordobil M. (2004). Relaciones de la socialización con inteligencia, autoconcepto y otros rasgos de la personalidad en niños de 6 años. Apunt. Psicol..

[19] Makri E., Giannouli V. (2022). Cross-cultural cognitive and affective differences in aging: Can culture shape the expression and perception of psychopathology in old age?. Encephalos.

[20] Tabernero C., Serrano A., Mérida R. (2017). Estudio comparativo de la autoestima en escolares de diferente nivel socioeconómico. Psicol. Educ..

[21] Garaigordobil M., Berrueco L. (2007). Efectos de un programa de intervención en niños de 5 a 6 años: Evaluación del cambio proactivo en factores conductuales y cognitivos del desarrollo. Summa Psicológica UST.

[22] Díaz L. Propuesta de intervención psicoeducativa para la elaboración de un programa de desarrollo de la autoestima y autoconcepto en el alumnado de Educación Infantil. Final Degree Project, Degree in Early Childhood Education, University of Zaragoza. ZAGUAN, Repositorio Institucional de Documentos. https://zaguan.unizar.es/record/120450.

[23] Ferreria M., Caeiro M., Marín R., Roldán J. (2020). Identidad y autoconcepto con niños y adolescentes. Aprendiendo a Enseñar Artes Visuales un Enfoque a/r/Tográfico.

[24] Franco C. (2018). Relajación creativa, creatividad motriz y autoconcepto en una muestra de niños de Educación Infantil. Rev. Electrónica Investig. Psicoeduc..

[25] Pulido E.G., Redondo M.P., Lora L.J., Jiménez L.K. (2023). Medición del Autoconcepto: Una revisión. Psykhe.

[26] Slutzky C.B., Simpkins S.D. (2009). The link between children’s sport participation and self-esteem: Exploring the mediating role of sport self-concept. Psychol. Sport Exerc..

[27] Cámara-Martínez A., Ruiz-Ariza A., Suárez-Manzano S., Cruz-Cantero R.M., Martínez-López E.J. (2023). Effect of an Integrated Active Lessons Programme through Playful Maths Games on Self-Concept, Self-Esteem and Social Skills in Preschool Children. Behav. Sci..

[28] Villa A., Auzmendi E. (2010). Desarrollo y Evaluación del Autoconcepto en la Edad Infantil.

[29] Aparicio L., Alcaide M. (2017). El autoconcepto en alumnos de Educación Infantil (3–6 años) según el género. Etic@net.

[30] Zavala R.A., Magallanes M.R. (2021). El impacto del autoconcepto académico de las niñas y los niños de preescolar en su desempeño académico, en el campo formativo de Lenguaje y Comunicación. Cretam.

[31] Harter S., Pike R. (1984). The Pictorial Scale of Perceived Competence and Social Acceptance for Young Children. Child Dev..

[32] Donolato E., Cardillo R., Mammarella E.C., Melby-Lervåg M. (2022). Research Review: Language and specific learning disorders in children and their co-occurrence with internalizing and externalizing problems: A systematic review and meta-analysis. J. Child Psychol. Psychiatry Allied Discip..

[33] Wilmot A., Pizzey H., Leitão S., Hasking P., Boyes M. (2023). Growing up with dyslexia: Child and parent perspectives on school struggles, self-esteem, and mental health. Dyslexia.

[34] Crisci G., Cardillo R., Mammarella I.C. (2022). The Processes Underlying Positive Illusory Bias in ADHD: The Role of Executive Functions and Pragmatic Language Skills. J. Atten. Disord..

[35] Erickson S., Bridgman K., Furlong L. (2023). Australian speech-language pathologists’ experiences and perceptions of working with children who stutter: A qualitative study. J. Fluen. Disord..

[36] Juárez Sánchez A., Monfort M. (2007). Registro Fonológico Inducido. Tarjetas Gráficas.

[37] Gallego Ortega J.L. (2012). Los trastornos de lenguaje en el niño. Estudios de Caso.

[38] Forns M., Triadó C., Forns M. (1989). Consideraciones acerca de la Evaluación del Lenguaje. La Evaluación del Lenguaje.

[39] Gallego Ortega J.L., Gómez Pérez I.A., Ayllón Blanco M.F. (2015). Evaluación de un programa dirigido a niños con trastorno fonológico. Rev. Investig. Logop..

[40] Alonso J., Román J.M. (2005). Prácticas educativas familiares y autoestima. Psicothema.

[41] Cohen L., Manion L., Morrison K. (2017). Research Methods in Education.

[42] Hoyle R., Duvall J. (2004). Determining the Number of Factors in Exploratory and Confirmatory Factor Analysis. The SAGE Handbook of Quantitative Methodology for the Social Sciences.

[43] Moafian F., Ostovar S., Griffiths M.D., Hashemi M. (2019). The construct validity and reliability of the ‘characteristics of successful efl teachers questionnaire (Coseflt-q)’ revisited. Porta Linguarum.

[44] Cobos Alvarado F., Peñaherrera León M., Ortiz Colon A.M. (2016). Validation of a questionnaire on research-based learning with engineering students. J. Technol. Sci. Educ..

[45] Deng L., Chan W. (2017). Testing the Difference between Reliability Coefficients Alpha and Omega. Educ. Psychol. Meas..

[46] Mcmillan J.H., Schumacher S. (2012). Research in Education: Evidence-Based Inquiry.

[47] Peters G. (2014). The alpha and the omega of scale reliability and validity: Why and how to abandon Cronbach’s alpha and the route towards more comprehensive assessment of scale quality. Eur. Heal Psychol..

[48] Hernández-Sampieri R., Mendoza Torres C.P. (2018). Metodología de la Investigación. Las Rutas Cuantitativa, Cualitativa y Mixta.

[49] Mondragón Barrera M.A. (2014). Uso de la correlación de Spearman en un estudio de intervención en fisioterapia. Mov. Científico.

[50] Cohen J. (1988). Statistical Power Analysis for the Behavioral Sciences.

[51] Maciej Tomczak E.T. (2014). The need to report effect size estimates revisited. An overview of some recommended measures of effect size. Trends Sport Sci..

[52] Ventura-León J.L. (2019). Tamaño del efecto para Kruskal-Wallis: Aportes al artículo de Domínguez-González et al. Investig. Educ. Médica.

[53] Jiménez Boraita R., Arriscado Alsina D., Gargallo Ibort E., Dalmau Torres J.M. (2022). Hábitos y calidad de vida relacionada con la salud: Diferencias entre adolescentes de entornos rurales y urbanos. An. Pediatría.

[54] Boerrigter M., Vermeulen A., Marres H., Mylanus E., Langereis M. (2021). Self-concept of children and adolescents with cochlear implants. Int. J. Pediatr. Otorhinolaryngol..

[55] Bernal E., Suquilanda P., Espinoza C., León A., Robles Y., Robles V., Quisi D. ISLanD: An informatics intelligent system to support the language development of children from 4 to 5 years. Proceedings of the IEEE International Autumn Meeting on Power, Electronics and Computing (ROPEC).

[56] Nevárez A., Reyes P., González C., Ortiz C., Nevárez M. (2019). Bucco-phonatory training practices with oral devices and immersive virtual reality: A pilot study. Rev. Chil. Fonoaudiol..

[57] Tenesaca O., León A., Velázquez V., Robles V. FonAPP: An Interactive Application for the Therapeutic Intervention of Children with Dyslalia from Embedded Devices and Robotic Assistants. Proceedings of the International Conference on Applied Human Factors and Ergonomics.

[58] Velásquez V., Mosquera K., Robles V., León A., Krupke V., Knox J., Torres V., Chicaiza P. An educational robotic assistant for supporting therapy sessions of children with communication disorders. Proceedings of the 7th International Engineering, Sciences and Technology Conference (IESTEC).

